# The Study of Dynamic Potentials of Highly Excited Vibrational States of DCP: From Case Analysis to Comparative Study with HCP

**DOI:** 10.3390/ijms17081280

**Published:** 2016-08-22

**Authors:** Aixing Wang, Chao Fang, Yibao Liu

**Affiliations:** 1 Fundamental Science on Radioactive Geology and Exploration Technology Laboratory, East China University of Technology, Nanchang 330013, China; xingxing_fz@sina.com (A.W.); liuyb01@mails.tsinghua.edu.cn (Y.L.); 2 School of Science, East China University of Technology, Nanchang 330013, China; 3 Institute of Nuclear and New Energy Technology, Tsinghua University, Beijing 100084, China; 4 Key Laboratory of Advanced Reactor Engineering and Safety of Ministry of Education, Beijing 100084, China; 5 Collaborative Innovation Center of Advanced Nuclear Energy Technology, Beijing 100084, China

**Keywords:** HCP, DCP, highly excited vibrational state, phase space, symmetry of dynamic potentials

## Abstract

The dynamic potentials of highly excited vibrational states of deuterated phosphaethyne (DCP) in the D–C and C–P stretching coordinates with anharmonicity and Fermi coupling are studied in this article and the results show that the D-C-P bending vibration mode has weak effects on D–C and C–P stretching modes under different Polyad numbers (*P* number). Furthermore, the dynamic potentials and the corresponding phase space trajectories of DCP are given, as an example, in the case of *P* = 30. In the end, a comparative study between deuterated phosphaethyne (DCP) and phosphaethyne (HCP) with dynamic potential is done, and it is elucidated that the uncoupled mode makes the original horizontal reversed symmetry breaking between the dynamic potential of HCP ($q_{3}$) and DCP ($q_{1}$), but has little effect on the vertical reversed symmetry, between the dynamic potential of HCP ($q_{2}$) and DCP ($q_{3}$).

## 1. Introduction

Resonance coupling between the different vibrational modes of molecules, which typically increases with energy, makes triatomic molecules quite intricate [1]. The ways of studying the resonance coupling effect between the different modes in a triatomic molecule are ab initio calculations and semi-classical methods [2,3,4]. In recent years, a new semi-classical method, named dynamics potential [5,6,7], has been proposed and has been applied to study highly excited molecular vibrational states. This method could, not only verify the conclusions given by ab initio calculations, but also show visual physical pictures, including molecular isomerization [8,9], chaotic dynamics [10,11], dissociation dynamics [12], and other information.

The internal interaction between D–C stretching and C–P stretching in DCP (deuterated phosphaethyne) has attracted a great deal of attention, since the information involved in the interaction is significant for understanding the mechanisms of chemical reactions. In previous articles, we analyzed the dynamic features of deuterated phosphaethyne (DCP) and phosphaethyne (HCP) using dynamic potentials [5]. Because of the drastic change of atomic masses of DCP compared with HCP, instead of the resonance between C–P stretching and D–C–P bending, a 2:1 D–C stretching and C–P stretching resonance governs the DCP spectrum. It is shown that there is dynamic symmetry between DCP and HCP systems, which is significant to analyze the features of homologous compounds.

In this work, the dynamic potentials of highly excited vibrational states and phase space trajectories of DCP are studied. The effect of the D–C–P bending vibration mode on the D–C and C–P stretching modes, under different Polyad numbers, are also investigated. Finally, a comparative study between DCP and HCP is done to clarify the symmetry breaking of dynamic potentials in DCP and HCP systems with the effects of uncoupled modes, respectively.

## 2. The Semi-Classical Hamiltonian of the DCP

The dynamic properties of DCP molecules’ highly-excited vibrational states, in the energy region 1.97 × 10^4^–2.35 × 10^5^ cm^−1^, are essential [5,9], and the corresponding Hamiltonian could be obtained as following:

(1) $$\begin{array}{ll} H & =\omega_{1}(n_{1}+\frac{1}{2})+\omega_{2}(n_{2}+\frac{1}{2})+\omega_{3}(n_{3}+\frac{1}{2})+X_{11}{\left(n_{1}+\frac{1}{2}\right)}^{2}\\ & +X_{12}(n_{1}+\frac{1}{2})(n_{2}+1)+X_{13}(n_{1}+\frac{1}{2})(n_{3}+\frac{1}{2})+X_{22}{\left(n_{2}+1\right)}^{2}\\ & +X_{23}(n_{2}+1)(n_{3}+\frac{1}{2})+X_{33}{\left(n_{3}+\frac{1}{2}\right)}^{2}+y_{111}{\left(n_{1}+\frac{1}{2}\right)}^{3}+y_{112}{\left(n_{1}+\frac{1}{2}\right)}^{2}(n_{2}+1)\\ & +y_{113}{\left(n_{1}+\frac{1}{2}\right)}^{2}(n_{3}+\frac{1}{2})+y_{122}(n_{1}+\frac{1}{2}){\left(n_{2}+1\right)}^{2}+y_{123}(n_{1}+\frac{1}{2})(n_{2}+1)(n_{3}+\frac{1}{2})\\ & +y_{133}(n_{1}+\frac{1}{2}){\left(n_{3}+\frac{1}{2}\right)}^{2}+y_{222}{\left(n_{2}+1\right)}^{3}+y_{222}(n_{2}+1)3+y_{223}{\left(n_{2}+1\right)}^{2}(n_{3}+\frac{1}{2})\\ & +y_{233}(n_{2}+1){\left(n_{3}+\frac{1}{2}\right)}^{2}+y_{333}{\left(n_{3}+\frac{1}{2}\right)}^{3}+z_{1111}{\left(n_{1}+\frac{1}{2}\right)}^{4}\\ & +z_{1112}{\left(n_{1}+\frac{1}{2}\right)}^{3}(n_{2}+1)+z_{1222}(n_{1}+\frac{1}{2}){\left(n_{2}+1\right)}^{3}+z_{1233}(n_{1}+\frac{1}{2})(n_{2}+1){\left(n_{3}+\frac{1}{2}\right)}^{2}\\ & +z_{2222}{\left(n_{2}+1\right)}^{4}+z_{2233}{\left(n_{2}+1\right)}^{2}{\left(n_{3}+\frac{1}{2}\right)}^{2}+z_{2333}(n_{2}+1){\left(n_{3}+\frac{1}{2}\right)}^{3}+z_{3333}{\left(n_{3}+\frac{1}{2}\right)}^{4}\\ & +[k+\lambda_{1}n_{1}+\lambda_{3}(n_{3}+\frac{3}{2})+\mu_{11}{n_{1}}^{2}](a_{3}^{+2}a_{1}+a_{3}^{2}a_{1}^{+}) \end{array}$$

The corresponding coefficients of the DCP Hamiltonian are shown in Table 1, where subscripts 1, 2, and 3, correspond to the D–C stretching vibration mode, D–C–P bending vibration mode, and C–P stretching vibration mode, respectively. We will use *n* to denote the corresponding vibration mode, which will be indicated with $q_{i}$ in the position coordinate, and the momentum coordinate indicated with $p_{i}$. $\omega_{i}$ is the corresponding harmonic vibration coefficient, while *X*_ij_, *y*_ijm_, *z*_ijmn_ denote the nonlinear coupling coefficients of different modes (*X*_ij_ ~ coefficient of the two nonlinear coupling modes, *y*_ijm_ ~ coefficient of the three nonlinear coupling modes, *z*_ijmn_ ~ coefficient of the four nonlinear coupling modes). *k*, λ_1_, λ_3_, and μ_11_ represent the Fermi resonance strength coefficient, with regard to the quantum numbers of the three vibrational modes. Besides $n_{2}$, there is another conserved action called Polyad number *P* = 2$n_{1}$ + $n_{3}$ (*P* number). Equation (1) is used to study the dynamic properties of highly excited vibrational states in the region of $n_{1}$ ≤ 4, *P* ≤ 30 [9].

The coset space SU(2)/U(1) [13,14] could be used as the representing space of Hamiltonian and it could be rewritten in the coordinates ($q_{1}$, $p_{1}$) indicates with semi-classical representations as follows:

(2) $$\begin{array}{ll} H(n_{2},q_{1},p_{1},P) & =\omega_{1}(\frac{p_{1}^{2}+q_{1}^{2}}{2}+\frac{1}{2})+\omega_{2}(n_{2}+1)+\omega_{3}(P-(p_{1}^{2}+q_{1}^{2})+\frac{1}{2})+X_{11}{\left(\frac{p_{1}^{2}+q_{1}^{2}}{2}+\frac{1}{2}\right)}^{2}\\ & +X_{12}(\frac{p_{1}^{2}+q_{1}^{2}}{2}+\frac{1}{2})(n_{2}+1)+X_{13}(\frac{p_{1}^{2}+q_{1}^{2}}{2}+\frac{1}{2})(P-(p_{1}^{2}+q_{1}^{2})+\frac{1}{2})+X_{22}{\left(n_{2}+1\right)}^{2}\\ & +X_{23}(n_{2}+1)(P-(p_{1}^{2}+q_{1}^{2})+\frac{1}{2})+X_{33}{\left(P-(p_{1}^{2}+q_{1}^{2})+\frac{1}{2}\right)}^{2}+y_{111}{\left(\frac{p_{1}^{2}+q_{1}^{2}}{2}+\frac{1}{2}\right)}^{3}\\ & +y_{112}{\left(\frac{p_{1}^{2}+q_{1}^{2}}{2}+\frac{1}{2}\right)}^{2}(n_{2}+1)+y_{113}{\left(\frac{p_{1}^{2}+q_{1}^{2}}{2}+\frac{1}{2}\right)}^{2}(P-(p_{1}^{2}+q_{1}^{2})+\frac{1}{2})+y_{122}(\frac{p_{1}^{2}+q_{1}^{2}}{2}+\frac{1}{2}){\left(n_{2}+1\right)}^{2}\\ & +y_{123}(\frac{p_{1}^{2}+q_{1}^{2}}{2}+\frac{1}{2})(n_{2}+1)(P-(p_{1}^{2}+q_{1}^{2})+\frac{1}{2})+y_{133}(\frac{p_{1}^{2}+q_{1}^{2}}{2}+\frac{1}{2}){\left(P-(p_{1}^{2}+q_{1}^{2})+\frac{1}{2}\right)}^{2}\\ & +y_{222}{\left(n_{2}+1\right)}^{3}+y_{223}{\left(n_{2}+1\right)}^{2}(P-(p_{1}^{2}+q_{1}^{2})+\frac{1}{2})+y_{233}(n_{2}+1){\left(P-(p_{1}^{2}+q_{1}^{2})+\frac{1}{2}\right)}^{2}\\ & +y_{333}{\left(P-(p_{1}^{2}+q_{1}^{2})+\frac{1}{2}\right)}^{3}+z_{1111}{\left(\frac{p_{1}^{2}+q_{1}^{2}}{2}+\frac{1}{2}\right)}^{4}+z_{1112}{\left(\frac{p_{1}^{2}+q_{1}^{2}}{2}+\frac{1}{2}\right)}^{3}(n_{2}+1)\\ & +z_{1222}(\frac{p_{1}^{2}+q_{1}^{2}}{2}+\frac{1}{2}){\left(n_{2}+1\right)}^{3}+z_{1233}(\frac{p_{1}^{2}+q_{1}^{2}}{2}+\frac{1}{2})(n_{2}+1){\left(P-(p_{1}^{2}+q_{1}^{2})+\frac{1}{2}\right)}^{2}+z_{2222}{\left(n_{2}+1\right)}^{4}\\ & +z_{2233}{\left(n_{2}+1\right)}^{2}{\left(P-(p_{1}^{2}+q_{1}^{2})+\frac{1}{2}\right)}^{2}+z_{2333}(n_{2}+1){\left(P-(p_{1}^{2}+q_{1}^{2})+\frac{1}{2}\right)}^{3}+z_{3333}{\left(P-(p_{1}^{2}+q_{1}^{2})+\frac{1}{2}\right)}^{4}\\ & +[k+\lambda_{1}(\frac{p_{1}^{2}+q_{1}^{2}}{2}）+\lambda_{3}(P-(p_{1}^{2}+q_{1}^{2})+\frac{3}{2})+\mu_{11}(\frac{p_{1}^{2}+q_{1}^{2}}{2}{）}^{2}]\sqrt{2}(P-(p_{1}^{2}+q_{1}^{2}))q_{1} \end{array}$$

With the coordinate ($q_{3},p_{3}$), the Hamiltonian can be written as:

(3) $$\begin{array}{ll} H(n_{2},q_{3},p_{3},P) & =\omega_{1}(\frac{P}{2}-\frac{p_{3}^{2}+q_{3}^{2}}{4}+\frac{1}{2})+\omega_{2}(n_{2}+1)+\omega_{3}(\frac{p_{3}^{2}+q_{3}^{2}}{2}+\frac{1}{2})+X_{11}{\left(\frac{P}{2}-\frac{p_{3}^{2}+q_{3}^{2}}{4}+\frac{1}{2}\right)}^{2}\\ & +X_{12}(\frac{P}{2}-\frac{p_{3}^{2}+q_{3}^{2}}{4}+\frac{1}{2})(n_{2}+1)+X_{13}(\frac{P}{2}-\frac{p_{3}^{2}+q_{3}^{2}}{4}+\frac{1}{2})(\frac{p_{3}^{2}+q_{3}^{2}}{2}+\frac{1}{2})+X_{22}{\left(n_{2}+1\right)}^{2}\\ & +X_{23}(n_{2}+1)(\frac{p_{3}^{2}+q_{3}^{2}}{2}+\frac{1}{2})+X_{33}{\left(\frac{p_{3}^{2}+q_{3}^{2}}{2}+\frac{1}{2}\right)}^{2}+y_{111}{\left(\frac{P}{2}-\frac{p_{3}^{2}+q_{3}^{2}}{4}+\frac{1}{2}\right)}^{3}\\ & +y_{112}{\left(\frac{P}{2}-\frac{p_{3}^{2}+q_{3}^{2}}{4}+\frac{1}{2}\right)}^{2}(n_{2}+1)+y_{113}{\left(\frac{P}{2}-\frac{p_{3}^{2}+q_{3}^{2}}{4}+\frac{1}{2}\right)}^{2}(\frac{p_{3}^{2}+q_{3}^{2}}{2}+\frac{1}{2})\\ & +y_{122}(\frac{P}{2}-\frac{p_{3}^{2}+q_{3}^{2}}{4}+\frac{1}{2}){\left(n_{2}+1\right)}^{2}+y_{123}(\frac{P}{2}-\frac{p_{3}^{2}+q_{3}^{2}}{4}+\frac{1}{2})(n_{2}+1)(\frac{p_{3}^{2}+q_{3}^{2}}{2}+\frac{1}{2})\\ & +y_{133}(\frac{P}{2}-\frac{p_{3}^{2}+q_{3}^{2}}{4}+\frac{1}{2}){\left(\frac{p_{3}^{2}+q_{3}^{2}}{2}+\frac{1}{2}\right)}^{2}+y_{222}{\left(n_{2}+1\right)}^{3}+y_{223}{\left(n_{2}+1\right)}^{2}(\frac{p_{3}^{2}+q_{3}^{2}}{2}+\frac{1}{2})\\ & +y_{233}(n_{2}+1){\left(\frac{p_{3}^{2}+q_{3}^{2}}{2}+\frac{1}{2}\right)}^{2}+y_{333}{\left(\frac{p_{3}^{2}+q_{3}^{2}}{2}+\frac{1}{2}\right)}^{3}+z_{1111}{\left(\frac{P}{2}-\frac{p_{3}^{2}+q_{3}^{2}}{4}+\frac{1}{2}\right)}^{4}\\ & +z_{1112}{\left(\frac{P}{2}-\frac{p_{3}^{2}+q_{3}^{2}}{4}+\frac{1}{2}\right)}^{3}(n_{2}+1)+z_{1222}(\frac{P}{2}-\frac{p_{3}^{2}+q_{3}^{2}}{4}+\frac{1}{2}){\left(n_{2}+1\right)}^{3}\\ & +z_{1233}(\frac{P}{2}-\frac{p_{3}^{2}+q_{3}^{2}}{4}+\frac{1}{2})(n_{2}+1){\left(\frac{p_{3}^{2}+q_{3}^{2}}{2}+\frac{1}{2}\right)}^{2}+z_{2222}{\left(n_{2}+1\right)}^{4}\\ & +z_{2233}{\left(n_{2}+1\right)}^{2}{\left(\frac{p_{3}^{2}+q_{3}^{2}}{2}+\frac{1}{2}\right)}^{2}+z_{2333}(n_{2}+1){\left(\frac{p_{3}^{2}+q_{3}^{2}}{2}+\frac{1}{2}\right)}^{3}+z_{3333}{\left(\frac{p_{3}^{2}+q_{3}^{2}}{2}+\frac{1}{2}\right)}^{4}\\ & +[k+\lambda_{1}(\frac{P}{2}-\frac{p_{3}^{2}+q_{3}^{2}}{4}）+\lambda_{3}(\frac{p_{3}^{2}+q_{3}^{2}}{2}+\frac{3}{2})+\mu_{11}(\frac{P}{2}-\frac{p_{3}^{2}+q_{3}^{2}}{4}{）}^{2}]\sqrt{\frac{P}{2}-\frac{p_{3}^{2}+q_{3}^{2}}{4}}(q_{3}^{2}-p_{3}^{2}) \end{array}$$

The semi-classical Hamiltonian, mentioned above, could further be used to obtain dynamic potentials, which are necessary for studying the dynamic nature of the DCP’s highly excited vibrational states.

## 3. The Dynamic Features of the Highly Excited Vibration States of DCP

Two main parts would be addressed in the following: (1) the influences of bending modes to D–C and C–P stretching modes; (2) the phase space trajectories for each energy levels in the dynamic potentials when *P* = 30 (as a case study); and (3) the comparative study between DCP and HCP in the sense of symmetry of dynamic potential.

### 3.1. The Dynamic Potentials Corresponding Typical Polyad Number with Different Quantum Number $n_{2}$

**Figure 1:**
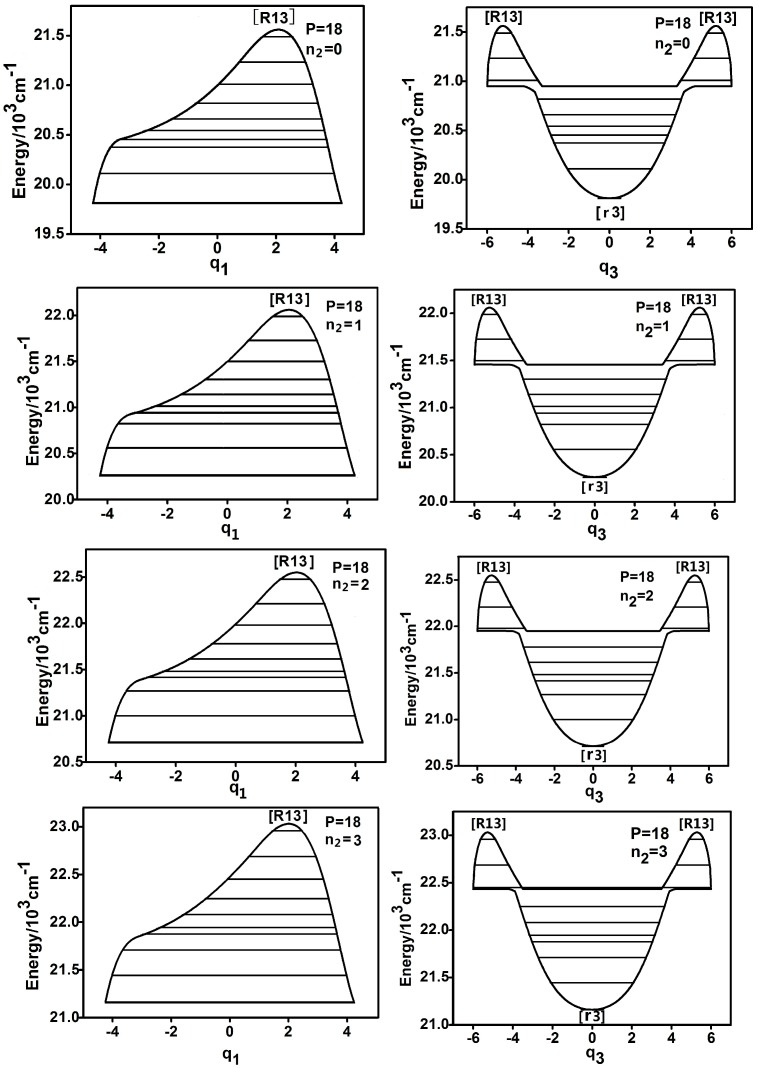
Dynamic potentials of DCP (*P* = 18) with $n_{2}$ = 0,1,2,3, and the energy levels included in the dynamic potential are shown by the lines.

The case of small *P* number will be firstly discussed. We take *P* = 18 as instance and the dynamic potential is shown as Figure 1 (the rule of marking fixed points is the same with the literature [5,6,9]). Figure 1 shows that when $n_{2}$ = 0,1,2,3, the dynamic potentials of $q_{1}$ coordinates are simple inverse Morse potential. It is known that corresponding to a certain *P*, the stability of the lowest energy level in an inverse Morse potential is the worst, while that of the highest one is the best, which is totally different from the concept in general potential that the lower the energy level is, the worse the stability is. Furthermore, the shapes of dynamic potentials of $q_{1}$ and $q_{3}$ coordinates under different $n_{2}$ are almost same, which elucidates that D-C-P bending has no effect on the stability of the highly excited vibrational states in DCP under the small P number. On the other hand, the dynamic potentials of $q_{3}$ show that the three modes corresponding to the highest three energy levels are localized and this conclusion is consistent when $n_{2}$ = 0,1,2,3. The dynamic potentials of $q_{1}$ and $q_{3}$ corresponding to different $n_{2}$ is basically the same and all the fixed points are remained when $n_{2}$ is different, which indicate that the effect of D-C-P bending mode has weak interaction with the two coupling modes, which are different from former studies of HOCl and HOBr systems [6,12].

**Figure 2:**
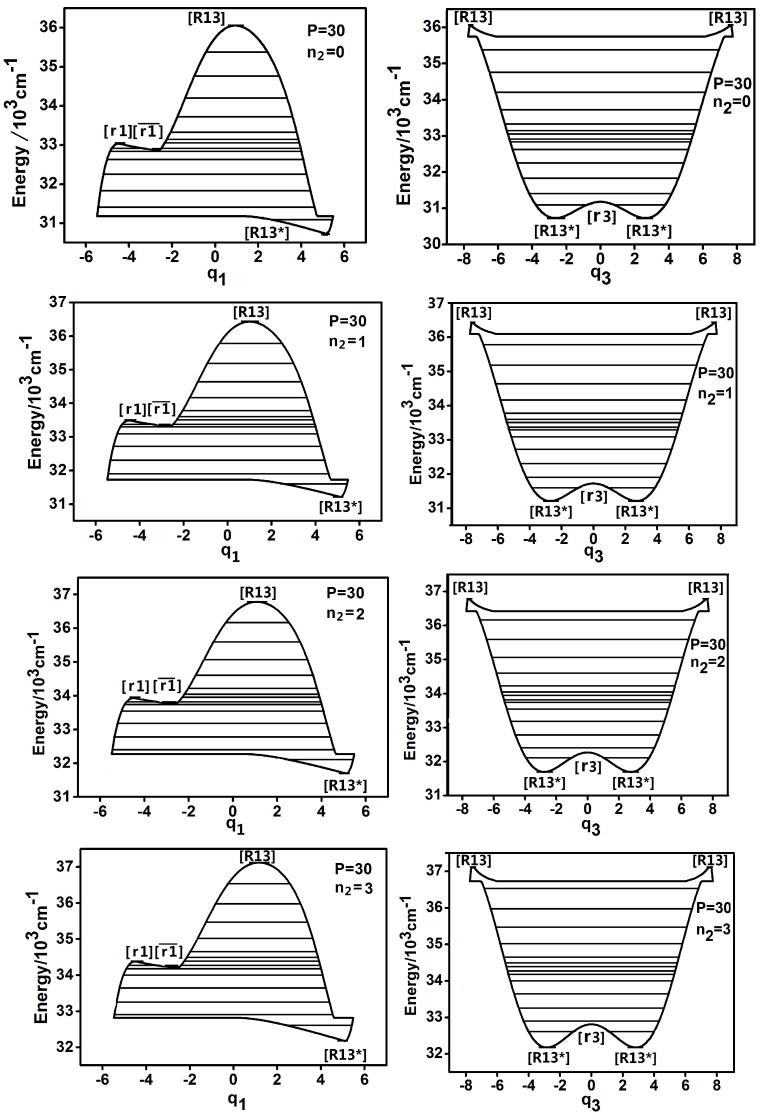
Dynamic potentials of DCP (*P* = 30) with $n_{2}$ = 0,1,2,3, and the energy levels included in the dynamic potential are shown by the lines.

Figure 2 shows the dynamic potentialins of $q_{1}$ and $q_{3}$ and it is shown that the results are different when P is large. For example, when *P* = 30, the dynamic potential of $q_{1}$ becomes much more complex. There are three new fixed points emerging, $\left[R_{13}*\right]$, $\left[r_{1}\right]$ and $\bar{\left[r_{1}\right]}$ in the dynamic potential and the shape of dynamic potential of $q_{1}$ becomes the combination of Morse and inverse Morse potentials. It is found that there is a phenomenon of fixed point-splitting in the dynamic potential of $q_{3}$ . The original $\left[r_{3}\right]$ (the dynamic potentialins of $q_{3}$ when *P* = 18 in Figure 1) becomes $\left[R_{13}*\right]$ and $\left[r_{3}\right]$ , which are similar with the results of HOBr and HOCl [6,12]. On the other hand, though the dynamic potentials of $q_{2}$ and $q_{3}$ when *P* = 30 are much more complex than the case of *P* = 18, but the shape of dynamic potentials of $q_{2}$ and $q_{3}$ remain the same when $n_{2}$ = 0,1,2,3, respectively, which is consistent with the case of *P* = 18.

Through the above study, it is found that the D–C–P bending mode weakly affects the resonant coupling of D–C and C–P stretching modes, thereby weakly affecting the dynamics features of DCP. It is shown that the geometrical shapes of the dynamic potentials and the corresponding fixed points are not sensitive to the D–C–P bending mode, but are sensitive to the *P* number, which are different to our previous studies [6,12]. Though the cases of *P* = 18 and *P* = 30 are shown here, these conclusions are also suitable for other cases. The reasons we address the cases of *P* = 18 and *P* = 30 are that the connotations of corresponding dynamic potentials are abundant and the shapes of dynamics potentials are typical.

### 3.2. The Trajectories of Phase Space Study for the Energy Levels under Specific Polyad Number (P = 30)

**Figure 3:**
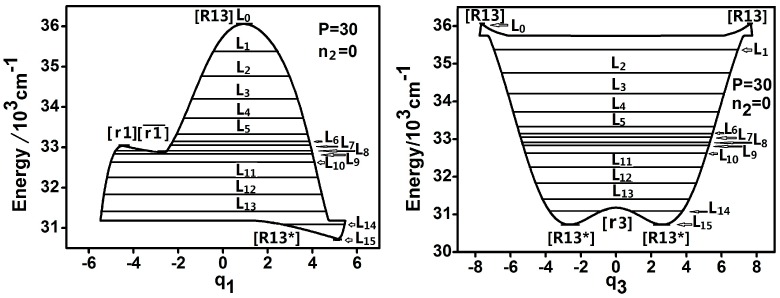
Dynamic potentials of DCP when *P* = 30 and $n_{2}$ = 0 and the energy levels included in the dynamic potential are shown by the lines.

For further quantitative analyzing the dynamic features of DCP, the representative trajectories of phase space in $p_{i}-q_{i}$ for each energy level is studied when *P* = 30. The dynamic potentials and corresponding energy levels when *P* = 30 and $n_{2}$ = 0 are shown in Figure 3.

**Figure 4:**
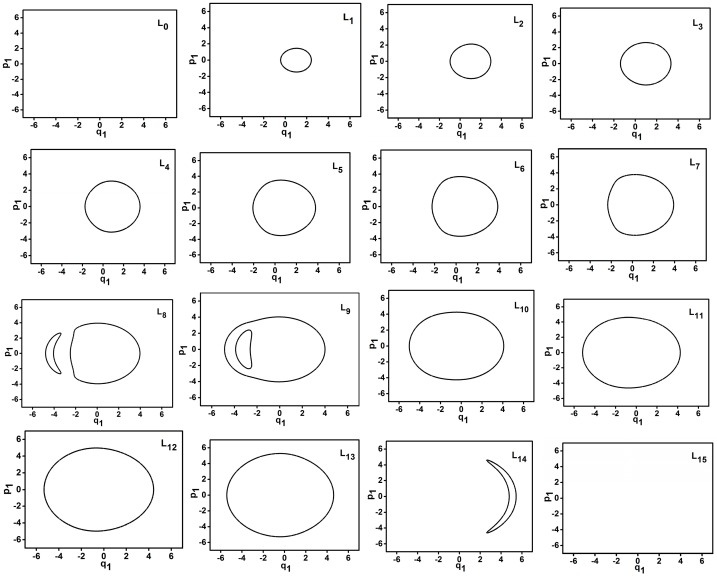
Trajectories of phase space of L0–L15 when *P* = 30 ($q_{1}$ coordinate).

**Figure 5:**
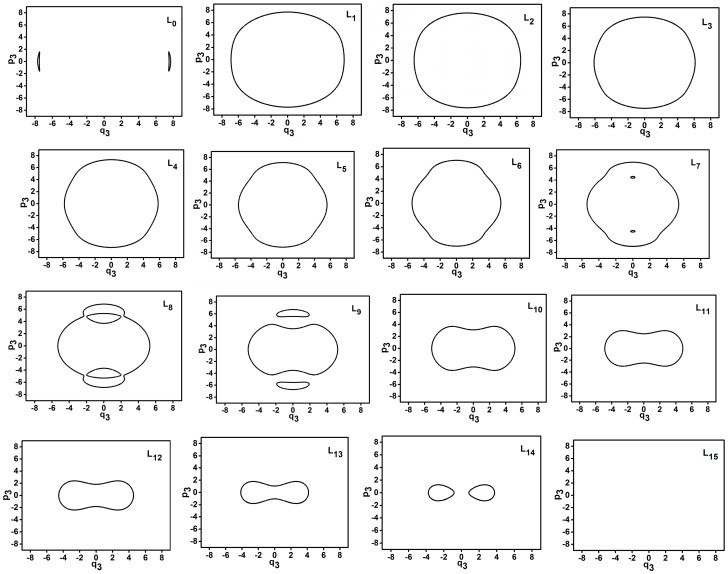
Trajectories of phase space of L0–L15 when *P* = 30 ($q_{3}$ coordinate).

The trajectories of phase space for different energy levels in dynamic potentials of $q_{1}$ and $q_{3}$ are shown in Figure 4 and Figure 5.

Based on previous studies, the envelope area of the trajectory in phase space shows the quantum environment of a series of energy levels [8,10]. In Figure 4, for L0–L13, it is found that the envelope area of the trajectory of phase space increases with the reduction of energy level because these energy levels lie in inverse Morse potential. In contrast, For L14–L15, the envelope area of the trajectory of phase space increases with the increase of energy level because these energy levels lie in Morse potential. Furthermore, because the L0 (L15) is tangential with the top (bottom) of the dynamic potential, the envelope area of the trajectory is zero. Particularly, the trajectories of L8 and L9 are divided into two separate trajectories, which show that these two energy levels are located in at a double-wells dynamic potential. The conclusions are similar in Figure 5. Because all energy levels (except L0 and L15) are in Morse potential of $q_{3}$ so the envelope area of the trajectory in phase space increases with the increase of energy level.

### 3.3. Comparative Study between DCP and HCP with Dynamic Potential

In previous work [5], it was shown that the dynamic potential of DCP in $q_{3}$ coordinate is similar to the inverse of that of HCP in $q_{2}$ coordinate and the dynamic potential of DCP in $q_{1}$ coordinate is similar to that of HCP in $q_{3}$ coordinate with $-q_{3}$ transformation, which is called “dynamic symmetry”. This conclusion is available when the quantum number of the uncoupling mode (H–C stretching mode for HCP and D–C–P bending mode for DCP) is equal to 0. However, the dynamic symmetry will be broken when the quantum number of the uncoupling mode ($n_{\text{un}}$, $n_{\text{un}}$ = $n_{1}$ for HCP and $n_{\text{un}}$ = $n_{2}$ for DCP [5]) is not equal to 0.

**Figure 6:**
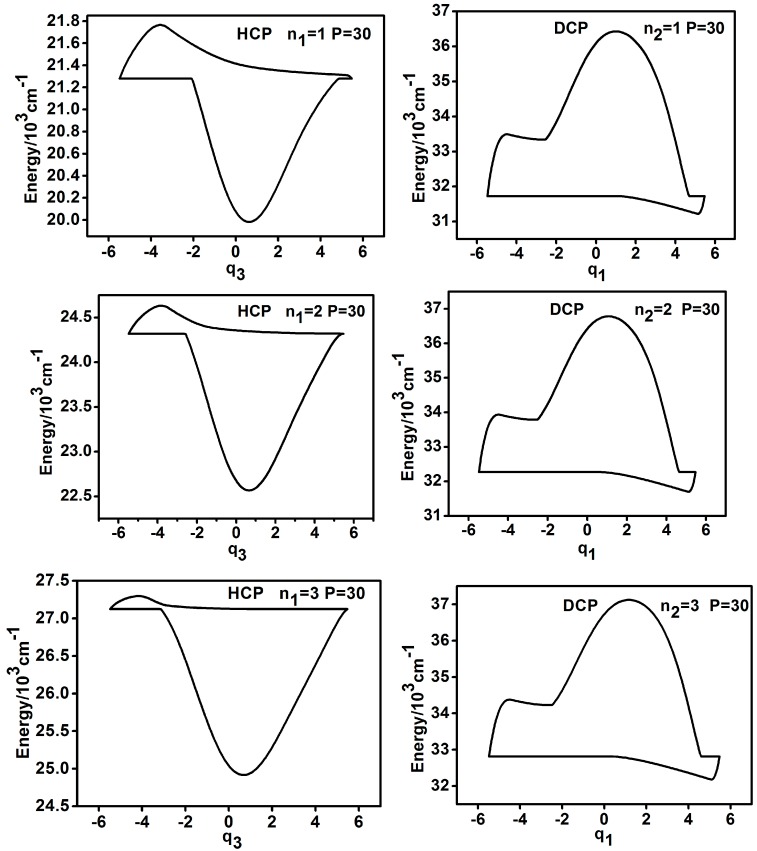
Dynamic potentials of HCP ($q_{3}$) and DCP ($q_{1}$) when $n_{\mathrm{un}}$ > 0 (*P* = 30).

**Figure 7:**
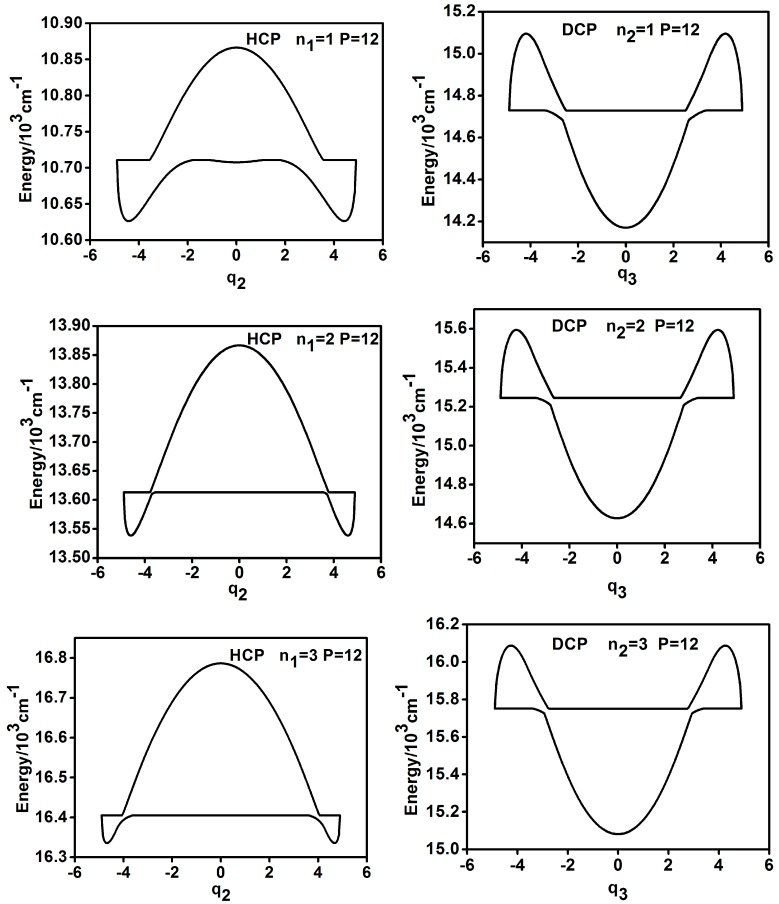
Dynamic potentials of HCP ($q_{2}$) and DCP ($q_{3}$) when $n_{\mathrm{un}}$ > 0 (*P* = 12).

From ergodic analysis of the P number, it is found that when $n_{\text{un}}$ > 0, the original horizontal reversed symmetry between the dynamic potentials of HCP ($q_{3}$) and DCP ($q_{1}$), mentioned in Reference [5], does not exist; however, for large *P* number (*P* > 22), the clockwise 180 °C rotation symmetry between the dynamic potentials of HCP ($q_{3}$) and DCP ($q_{1}$) emerge. This symmetry is not strict but it still could be recognized from the shapes of dynamic potentials and the above conclusion is consistent for $n_{\text{un}}$ = 1, 2, 3 (as shown in Figure 6, for instance). In contrast, the results of the dynamic potentials of HCP ($q_{2}$) and DCP ($q_{3}$) are different. As shown in Figure 7, for instance, the vertical reversed symmetry could remain for a small *P* number (10 < *P* < 20) but it is broken when *P* becomes large.

From the above results, it is shown that the uncoupled mode has an effect on the dynamic symmetry. It is obvious that the $n_{\text{un}}$ makes the original horizontal reversed symmetry break between the dynamic potential of HCP ($q_{3}$) and DCP ($q_{1}$) but has little effect on the vertical symmetry breaking between the dynamic potential of HCP ($q_{2}$) and DCP ($q_{3}$). It is elucidated that the stability of dynamic symmetry is different under the effect of the uncoupling mode.

## 4. Conclusions

In this study, the dynamic potentials of highly excited vibrational states of DCP with an harmonicity and Fermi coupling are studied. The results show that the D–C–P bending mode has weak effects on D–C and C–P stretching mode under different Polyad numbers. Just like previous studies, it is found that the vibrational energy levels could be classified by the quantum environments. From comparative studies, it shows that the uncoupled modes make the original horizontal reversed symmetry breaking between the dynamic potential of HCP ($q_{3}$) and DCP ($q_{1}$), but has little effect on the vertical symmetry between dynamic potential of HCP ($q_{2}$) and DCP ($q_{3}$). Considering the effect of $n_{2}$ in DCP and $n_{1}$ in HCP, the original dynamic similarities in these two systems disappear and the characteristics of symmetry become much more complex. The above results show that the method which enables us to understand the DCP dynamics simply from those of HCP without repeated elaboration are only available in some special conditions, and, on the other hand, there are some new dynamic symmetries appearing when the conditions are different, which indicate that the homologous compounds are intrinsically similar only if the coupling patterns of two systems are analogous.

## Figures and Tables

**Table 1:** The coefficients of vibration Hamiltonian of deuterated phosphaethyne (DCP).

Parameter Name	Parameter Values (cm^−1^)	Parameter Name	Parameter Values (cm^−1^)
$\omega_{1}$	2494.0412	*y*_223_	0.0482
$\omega_{2}$	539.1611	*y*_233_	0.2535
$\omega_{3}$	1237.0955	*y*_333_	−0.2447
*X*_11_	−24.0769	*z*_1111_	0.0510
*X*_12_	−11.1041	*z*_1112_	0.0806
*X*_13_	−4.6276	*z*_1222_	−0.0055
*X*_22_	−3.5142	*z*_1233_	0.0280
*X*_23_	−2.2082	*z*_2222_	−0.0014
*X*_33_	−2.2082	*z*_2233_	−0.0067
*y*_111_	−0.8896	*z*_2333_	−0.0132
*y*_112_	−0.4928	*z*_3333_	0.0092
*y*_113_	−0.5407	*k*	12.3422
*y*_122_	0.2167	λ_1_	0.5786
*y*_123_	−0.3655	λ_3_	0.1212
*y*_133_	−0.2167	μ_11_	−0.2990
*y*_222_	0.0884		
